# 247. Evaluation of the Efficacy and Safety of Cefazolin in Non-Pneumococci Streptococcal Bacteremia

**DOI:** 10.1093/ofid/ofad500.320

**Published:** 2023-11-27

**Authors:** Arya Wibisono, Dusten T T Rose, Theresa (Terry) Jaso

**Affiliations:** Ascension Seton, Austin, Texas; Dell Seton Medical Center at the University of Texas, Austin, Texas; Ascension Seton, Austin, Texas

## Abstract

**Background:**

Among the large variation of streptococcal infections, recommended therapies are generally well-defined. There is a lack of efficacy data on alternative therapies, notably in phylogenetic groups that are less studied. The purpose of this study is to quantify the differences in clinical efficacy and safety between recommended antibiotics (penicillins and ceftriaxone) compared to cefazolin for uncomplicated non-pneumococci streptococcal bacteremia.

**Methods:**

This is a multi-center, retrospective cohort study from January 2017 to January 2021. Patients were included if older than 18 years of age and had a non-pneumococci streptococcal isolate recovered on blood cultures. Patients were excluded if they had a polymicrobial bacteremia, diagnosed meningitis or endocarditis, received less than two days of a study drug, or received more than three days of a non-study drug. The primary outcome was treatment failure defined as: 30-day in-hospital all-cause mortality; or the alteration of targeted therapy after at least 48 hours of treatment for (1) concern of clinical failure based on progress notes in the electronic medical record or (2) an adverse drug reaction. Secondary outcomes included 90-day all-cause mortality, intensive care unit length of stay, hospital length of stay, *C. difficile* infection, adverse events, and 6-month infection related readmissions.

**Figure:**
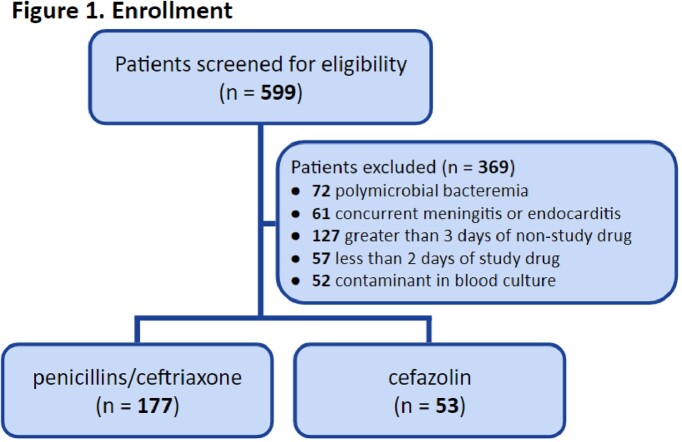

**Results:**

A total of 230 patients were included: 177 in the penicillins/ceftriaxone group and 53 in the cefazolin group. Baseline demographics were balanced between the two groups in regard to age, sex, comorbidity score, infection severity, and infectious disease consultation. The most commonly identified pathogens were *S. pyogenes* (30.9%), *S. agalactiae* (26.5%), and *S. mitis/oralis* (11.7%). Analysis showed no significant difference between treatment failures with recommended antibiotics (6/177, 3.4%) and cefazolin (1/53, 1.9%), p=1.00. No differences were noted in secondary safety outcomes.

**Figure:**
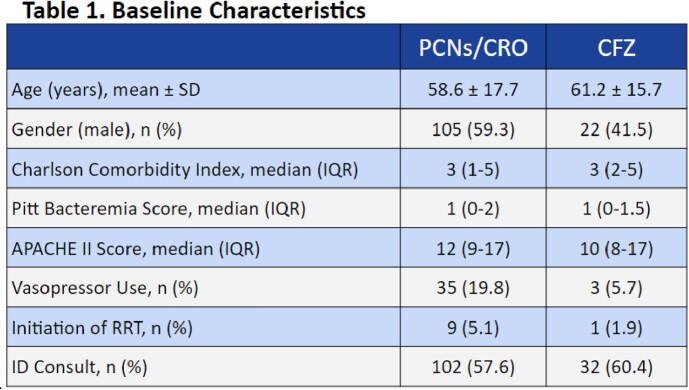

**Figure:**
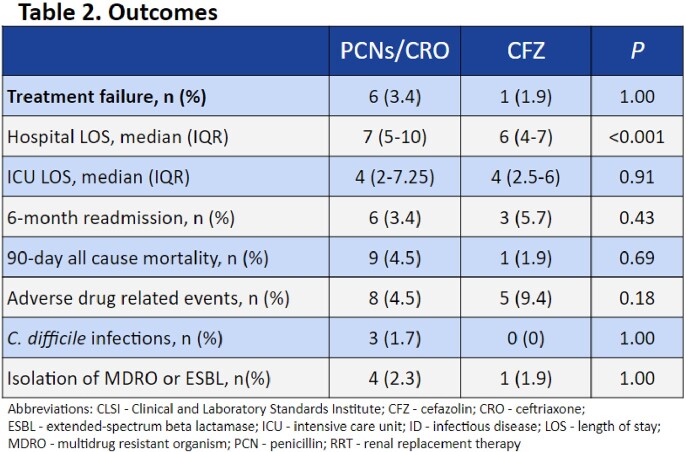

**Figure:**
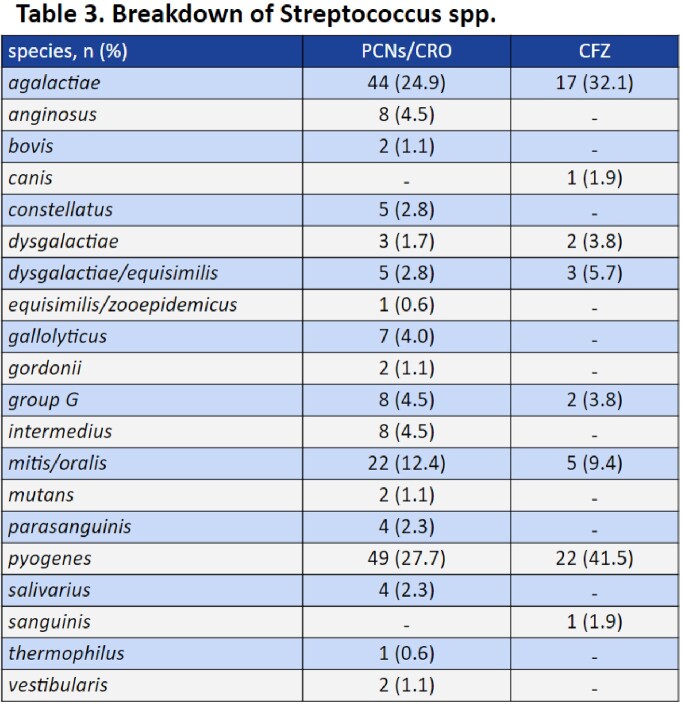

**Conclusion:**

Rates of treatment failure with cefazolin were not significantly different compared to recommended antibiotics in the treatment of uncomplicated non-pneumococci streptococcal bacteremia. Cefazolin may be a potential option for treatment in these infections. Further studies are warranted.

**Disclosures:**

**All Authors**: No reported disclosures

